# Hypoxia enhances autophagy level of human sperms

**DOI:** 10.1038/s41598-024-59213-1

**Published:** 2024-04-11

**Authors:** Jie Hu, Jiwei Wu, Xinge Liu, Yan Zhang, Linfeng Mo, Liangzhao Liu, Shengxue Liu, Chaoyan Ou, Yonghua He

**Affiliations:** 1 School of Public Health, Guilin Medical University, Zhiyuan Road, Lingui District, Guilin, 541199 Guangxi China; 2 Medicine and Health Science College, Guangzhou Huashang Vocational College, Guangzhou, 511300 Guangdong China; 3 Centre of Reproductive Medicine, Affiliated Hospital of Guilin Medical University, Yiwu Road, Xiufeng District, Guilin, 541001 Guangxi China

**Keywords:** Spermatozoa, Hypoxia-inducible factor-1α, Autophagy, Proteomics, Asthenozoospermia, Biochemistry, Cell biology, Molecular biology

## Abstract

The relationship between oxygen sensing and autophagy in human sperms was explored in this study. Health semen and asthenozoospermia (astheno) semen were incubated with hypoxia-inducible factor-1α (HIF-1α) interferents, i.e., lificiguat (YC-1) or cobalt chloride (CoCl_2_), respectively. Label-free quantitative proteomic technology was used to identify the differentially expressed proteins in human semen under the hypoxia condition. Selected proteins were detected with ELISA. It was found that the autophagy levels of sperm in the YC-1 + health group or CoCl_2_ + astheno group increased while the vitality decreased. A total of 17, 34 and 35 differentially expressed proteins were observed in the Astheno group, the YC-1 + health group and the CoCl_2_ + astheno group, respectively. These proteins were primarily associated with protein processing in endoplasmic reticulum, Th17 cell differentiation, progesterone-mediated oocyte maturation, glycolysis/gluconeogenesis, HIF-1 signaling pathway, biosynthesis of amino acids, and carbon metabolism. The expression levels of protein HIF-1α, LC3B, histone H4, cathepsin L and ENO1 changed significantly in the groups. The study suggests that hypoxia can increase sperm autophagy level and reduce their vitality through HIF-1 signaling pathway and glycolysis/gluconeogenesis signaling pathway. Furthermore, proteins histone H4, cathepsin L, glutathione synthetase and ENO1 are proposed as potential biomarkers of autophagy and vitality in asthenozoospermia sperm.

## Introduction

Human semen quality has been declining for several decades, with a notable decrease in sperm motility being a critical indicator. Asthenozoospermia (also known as asthenospermia, Astheno) is a condition characterized by the reduced progressive motility of sperm, which makes it challenging for sperm to reach and fertilize eggs. Astheno accounts for 20–40% of male infertility cases^1^. The decrease in sperm motility can be attributed to various congenital and acquired factors, and the mechanisms behind it are complex and not fully understood.

Oxygen sensing is a crucial biological process in cells, and hypoxia-inducible factor-1α (HIF-1α) plays a vital role in this process. Hypoxia triggers the activation of HIF-1, which leads to the transcription of hypoxia-related genes^2^. These genes regulate various cellular processes, including growth, proliferation, apoptosis, and survival^3–5^. Studies have demonstrated that in testicular tissue of male rats exposed to low air/oxygen pressure environment, the levels of HIF-1α protein and mRNA were significantly up-regulated^6–8^. This up-regulation resulted in reduced male reproductive function, as well as decreased sperm quantity and quality^9–11^. Similar studies conducted on human case of low sperm quality, compared with matched controls, have provided evidences of an association between down-regulated expression of autophagy-regulating genes and genes associated with low sperm quality^12^.

Oxygen sensing is closely linked to autophagy^13^, the process of cellular metabolism and renewal. Normally, autophagy operators at low level to maintain cellular homeostasis^14,15^. However, studies have observed significantly higher levels of HIF-1α, the key protein involved in oxygen sensing, in infertile men with astheno compared to fertile men. Furthermore, there is a negative correlation between expression of HIF-1α and sperm motility^16^. The growth and vitality of germ cells have been found to be negatively influenced by increased autophagy levels^17,18^. Multiple metabolic pathways, particularly glycolysis, are involved in this process^19^. Although the relationships between hypoxia and autophagy are well known in many health conditions, the specific mechanisms underlying the process remain unclear.

In this study, we hypothesized that HIF-1α might regulate autophagy levels and impact vitality of human sperm. To investigate this, we utilized label-free quantitative proteomic technology and incubated healthy or astheno semen samples with HIF-1α interferents. By examining the relationship between hypoxia and autophagy in human sperms, this study aims to shed light on the mechanism underlying sperm motility. Additionally, it may uncover potential biomarkers that could serve as novel targets for the diagnosis and treatment of astheno at protein level.

## Methods

### Study population

The study participants were randomly selected form individuals who visited the Centre of Reproductive Medicine, Affiliated Hospital of Guilin Medical University, between June 2021 and December 2022. The participants consisted of individuals with health semen quality and patients diagnosed with astheno. To ensure comparability, the two categories were matched based on age frequency.

Both the health semen samples and the astheno semen samples met the following criteria: (1) Semen volume greater than 1.5 mL; (2) Semen pH value ranging from 7.2 to 7.8; (3) Sperm concentration equal to or higher than 15 × 10^6^ cell/mL; (4) Liquefaction time of no more than 30 min; (5) Color of the semen: gray-white or light yellow; (6) Total sperm motility, which includes progressive (PR) and non-progressive (NP) motility, of the health semen had to be greater than 40%, with PR motility equal to or greater than 32%. In contrast, the astheno group had PR motility less than 32%. The inclusion criteria for the study were as the followings: (1) Males between the ages of 18 and 45; (2) No history of severe or significant environmental exposures such as heavy smoking, exposure to chemicals, or pesticides. The exclusion criteria were as the followings: (1) Individuals with a history of severe reproductive system trauma; (2) Long-term use of steroid drugs or medicines that affected sperm function; (3) Individuals with severe necrospermia, azoospermia, or cryptorchidism.

A questionnaire was used on-site to collect general and clinical data of the subjects, including name, age, smoking, drinking (alcohol consumption), medication history, sampling time, and abstinence days.

### Semen sample collection and treatment

The semen samples were collected through masturbation after abstinence of 3–7 days. The samples were kept warm at a temperature between 20 and 37 ℃ and were tested within 1 h. Semen is a mixture of sperm and secretions from the male reproductive organs. Sperm accounts for about 5–10% of the total ejaculate volume, while the remaining 90–95% (referred to as seminal plasma) primarily consists of water, proteins, polypeptides, carbohydrates, enzymes, inorganic salts, and organic small molecules. These components play crucial roles in sperm function and offer potential biomarkers for assessing male fertility. The testicular barrier effectively isolates and maintain the stability and specificity of seminal plasma components. Therefore, complete semen samples, which include both seminal plasma and sperm, were used for semen quality analysis and proteomics analysis in this study.

Soluble guanylate cyclase (sGC) activator (lificiguat, YC-1, Selleckchem, USA) has been shown to completely suppress the expression of HIF-1α under hypoxia condition^20^. On the other hand, Cobalt chloride (CoCl_2_, Mackin, China), a chemical hypoxia mimetic, can create a hypoxic environment and induce the accumulation of HIF-1α, inducing hypoxic damage in various cell models in vitro^21^. In this study, YC-1 and CoCl_2_ were used to incubate human semen samples to explore the effects of HIF-1α on autophagy and elucidate the underlying mechanisms.

A total of 85 qualified semen samples were included in the study and divided into 4 groups. The 41 samples of health semen were further randomly categorized into a health control group (Health, 20 samples) and a health experimental group (YC-1 + health, 21 samples). Similarly, the 44 samples of asthenozoospermia semen were divided into astheno control group (Astheno, 22 samples) and astheno experimental group (CoCl_2_ + astheno, 22 samples). According to the results of our pilot study, the semen samples of YC-1 + health group or that of the CoCl_2_ + astheno group were incubated with 5000 μM YC-1 or 100 nM CoCl_2_ at 37 ℃ for 60 min, respectively. Health group and Astheno group were not given any of the reagents.

### Sperm motility assay

The semen was gently and thoroughly mixed, and then 0.1 mL of the sample was applied on a slide. A computer-aided sperm analysis system (CASA, ZJ-3000E, Xuzhou, China) was used to analyze sperm concentration and PR (%).

### MDC determination for autophagy level of sperms

#### Semen sample purification

The SWIM-UP method was employed to purify the semen samples. Briefly, a total of 2 mL preheated fallopian tube fluid (HTF, MCE, USA) at 37 ℃ human and 500 μl semen sample were separately added to a centrifuge tube. A clear boundary line between the semen and HTF solution was created so as to segregate the sperms based on their motility. The centrifuge tube was tilted at 45° angle and incubated at 37 ℃ for 1 h.

#### Experimental procedures of MDC method

Following the instructions of the MDC (Monodansylcadaverin) autophagy staining test kit (Solarbio, China), 875 μl of the supernatant was collected and centrifuged at 700*g* for 5 min. The cells were dispersed with 1 × Wash buffer and centrifuged at 800 g for 5 min to collect the cells. The cells were re-suspended in 1 × Wash buffer, counted, and adjusted to a concentration of 1 × 10^6^ cell/mL. A volume of 90 μl of the cell suspension was added into an EP tube and stained with 10 μl of MDC for 30 min in darkness. The cells were then washed with 400 μl of 1 × Wash buffer and centrifuged. This washing process was repeated twice. The cells were finally re-suspended in 100 μl of collection buffer. A volume of 10 μl of the cell suspension was plated on a glass slide, covered, and observed using a fluorescence microscope (Leica, Germany) with an excitation filter wavelength of 355 nm and a blocking filter wavelength of 512 nm. The autophagy level of the sperm was calculated according to the instructions provided with the kit.

### Label-free proteomic experiments

#### Protein extraction and digestion

The semen sample was combined with lysis buffer (Promega, US) and sonicated for 5 min in an ice bath. The lysis buffer consisted of 8M urea (GibcoBRL, US), 30 mM HEPES (AMRECSO, US), 1 mM PMSF (AMRECSO, US), 2 mM EDTA (AMRECSO, US) and 10 mM DTT (Promega, US). Subsequently, the samples were centrifuged at 20,000*g* for 30 min. The supernatant, along with 10 mM DTT, was incubated at 56 ℃ for 1 h and then mixed with 55 mM IAM (Promega, US), followed by incubation in darkness for 1 h. After centrifugation at 20,000*g* and 4 ℃ for 30 min, the protein concentration was determined using the Bradford method.

Once protein extraction was completed, digestion was performed. 40 μg of protein from each sample was transferred to a 3K ultrafiltration tube and centrifuged at 14,000*g* and 4 ℃ for 40 min. Then, 200 μl of 50 mM NH_4_HCO_3_ was added to the sediments and centrifuged again. This process repeated twice. Next, 1 μg/μL trypsin was added to the sample at a protein substrate to enzyme ratio of 30:1, and the mixture was incubated in a water bath at 37 ℃ for 24 h. After the digestion solution was lyophilized, the peptide segments were re-suspended in 30μL 25 mM NH_4_HCO_3_.

### Mass spectrometry detection and data processing

The Q-Exactive mass spectrometer (Thermo Q Exactive™, US) was utilized to detect the peptide signals of each purified sample. The liquid phase and mass spectrometry detection parameters were as follows:

Mass spectrometry detection-nanoliter LC column and mobile phase: NanoLC trap: Acclaim PePmap 100, 150 μm × 2 cm nanoviper C18 5 μm 100Å; NanoLC column: C18 5μm 75 μm × 15 cm aperture 300Å; Solvent A: 0.1% formic acid 2% CAN 98% water; Solvent B: 0.1% formic acid (Huayi, China) 2% water 98% CAN (Fisher Scientific, US); Flow rate: 0.4 μl/min.

Mass spectrometry detection-nanoLC liquid phase gradient: B liquid ratio and time: 2% (0 min), 2% (6 min), 35% (95 min), 95% (105 min), 95% (115 min), 2% (115.1 min), 2% (120 min).

Mass spectrometry detection-Q-E mass spectrometer parameters: Polarity: positive ion mode, MS scan range: 350–1350 m/z, Resolution: 60,000, Capillary temperature: 350 degrees, Ion source voltage: 2200 V, MS/MS acquisition modes: Higher collision energy dissociation (HCD), Normalized collision energy (NCE): 38.

After mass spectrometry scanning, the mass spectrometry raw file was obtained. MaxQuant software (https://www.maxqda.com/, version: 1.6.0.1) was applied for data input, screening and quantitative analysis of the raw files. The screening parameters, retrieval and quantitative parameters are detailed below.

Spectrum screening parameters: precursor ion mass range: 350–6000 Da; minimum peak number in MS/MS spectrum: 10; signal-to-noise ratio (S/N threshold): 1.5.

Identification retrieval parameters: MaxQuant; Fixed modification: Carbamidomethyl (C); Variable modification: Oxidation (M), Gln → Pyro-Glu (N-term Q); Peptide tol: 15ppm; MS/MS tol: 20mmu; Max missed cleavages: 1; Enzyme: Trypsin; Database: Uniprot_human, Number of sequences: 204,703.

Quantitative parameters: Protein ratio type and Normalisation method: Intensity; Minimum peptides: 1.

The significance of protein differences was evaluated using MSstats variance analysis in R software (http://www.rrsoftware.com/, version 4.1.0). The criteria for screening differentially expressed proteins were as follows: (1) The fold change in protein average abundance was greater than 1.2 or less than 0.83, and (2) The *p*-value of T-test was less than 0.05.

### Detection of related protein using ELISA

The semen sample was adjusted to a cellular concentration of 1 × 10^6^ cell/mL with PBS (pH 7.2–7.4). After undergoing several cycles of freezing and thawing, 0.5 mL of lysis solution was added to lyse the cells on ice. The mixture was then centrifuged to collect the proteins, and the concentration of total protein was measured with Bradford method.

The levels of glutathione synthetase in the semen samples were determined using the Human Glutathione Synthetase ELISA Kit (Ruichuang, Shanghai, China). Following the kit’s instructions, varied concentrations of standard samples (50 μL each) were added into the standard wells. Blank wells (without samples and enzyme conjugate) and test sample wells were also prepared. In the test sample wells on the coated plate, 40 μL of sample diluent was added first, followed by the addition of 10 μL of the test sample (resulting in a final dilution factor of 5). The samples were placed at the bottom of the plate wells, ensuring no contact with the well walls, and gently mixed. Enzyme conjugate (100 μL) was added to each well (except the blank ones). The plate was sealed with a sealing film and incubated at 37°C for 60 min. After carefully removing the sealing film, the liquid was discarded, and the plate was tapped to remove water droplets. Each well was filled with washing buffer, and then dried. Next, 50 μL of reagent A and 50 μL of reagent B were added in each well. The plate was gently shaken to mix the contents and incubated at 37 °C in darkness for 15 min for color development. Subsequently, 50 μL of stop solution was added to terminate the reaction (turning the color from blue to yellow). The absorbance (OD value) was measured at a wavelength of 450 nm, with the blank well used for zero calibration. The measurement was performed within 15 min after adding the stop solution. The concentrations of the samples were calculated using the linear regression curve equation derived from the standard samples.

Similar operations were followed for the detection of HIF-1α (Jinpin, Shanghai, China), LC3B (Chutai, Shanghai), ENO1 (Xinweiyu, Shanghai,), PGK1 (spbio, Wuhan, China), Cathepsin L (balb, Beijing), and Histone H4 (FineTest, Wuhan, China) using their respective ELISA kits.

### Statistical analysis

SPSS 25.0 and GraphPad Prism 8.0 were used for data analysis. The normality test was performed on various basic information variables of all semen samples, including age, abstinence days, sperm concentration, sperm PR, sperm autophagy, and expression of related proteins. Based on the distribution characteristics of the data, multiple linear regression was applied to analyze the relationship between sperm forward motility and related factors. Other statistical methods included analysis of variance (ANOVA), paired sample t-test, rank sum test, chi-square test.

### Ethics approval and consent to participate

This study was approved by the Medical Ethics Committee of Affiliated Hospital, Guilin Medical University. Signed informed consents were obtained from the subjects.

All methods were carried out in accordance with relevant guidelines and regulations.

## Results

### General and clinical data analysis

A total of 85 qualified semen samples were included in the analysis, with 41 samples obtained from health individuals and 44 samples from astheno patients. None of the subjects had a history of relevant medicine use. The prevalence of smoking and drinking of the populations were not statistically different between the groups. There was no significant correlation between PR and factors such as age, interval of sample collection and assessment, abstinence days, and sperm concentration (Table 1).

**Table 1:** General and clinical data of the subjects. Astheno is Asthenozoospermia. Values are mean ± SD or case number (percentage).

	Health semen (n = 41)	Astheno semen (n = 44)	P-value
Age (years)^a^	33.68 ± 3.52	32.91 ± 5.06	> 0.05
Body mass index (kg/m^2^)^a^	22.62 ± 1.64	22.48 ± 1.34	> 0.05
Smoking (n, %)^b^	12 (29.3)	13 (29.5)	> 0.05
Drinking (n, %)^b^	16 (39.0)	18 (40.9)	> 0.05
Abstinence days (days)^a^	4.34 ± 1.26	4.43 ± 1.65	> 0.05
Interval of sample collection and assessment (min)^b^	65.88 ± 22.64	63.08 ± 45.71	> 0.05
Sperm concentration (× 10^6^ cell/mL)^c^	98.40 ± 46.51	111.78 ± 86.76	> 0.05
Progressive sperm rate (%)^a^	63.03 ± 14.37	23.35 ± 6.26	< 0.01

^a^Analysis of variance (ANOVA) has been used.

^b^Chi-square test has been used.

^c^Rank sum test has been used.

### Effect of hypoxia on sperm vitality

**Figure 1:**
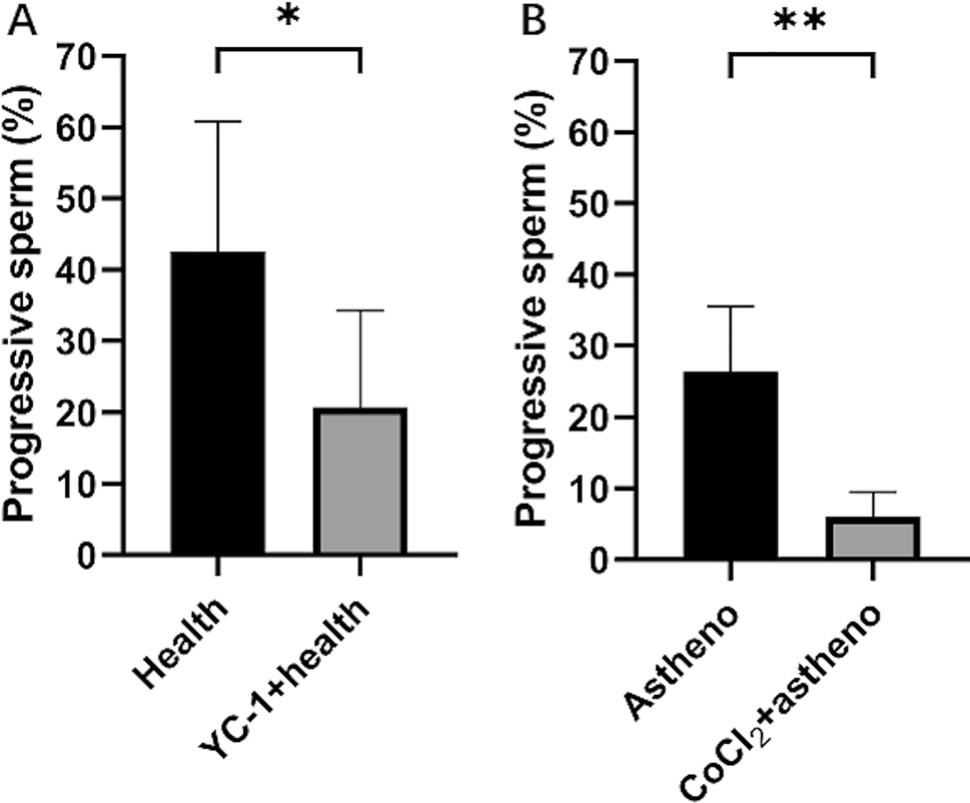
Effect of incubation in 5000 μM YC-1 (**A**, ^*^p < 0.05) or 100 nM CoCl_2_ (**B**, **p < 0.01) at 37 ℃ for 60 min on sperm rate of progressive motility assayed with computer-aided sperm analysis system. Astheno is Asthenozoospermia. Analysis of variance (ANOVA) has been used.

After incubation with YC-1 or CoCl_2_ for 60 min, PRs were significantly reduced compared with the corresponding control groups (Fig. 1).

### Effect of hypoxia on sperm autophagy level

**Figure 2:**
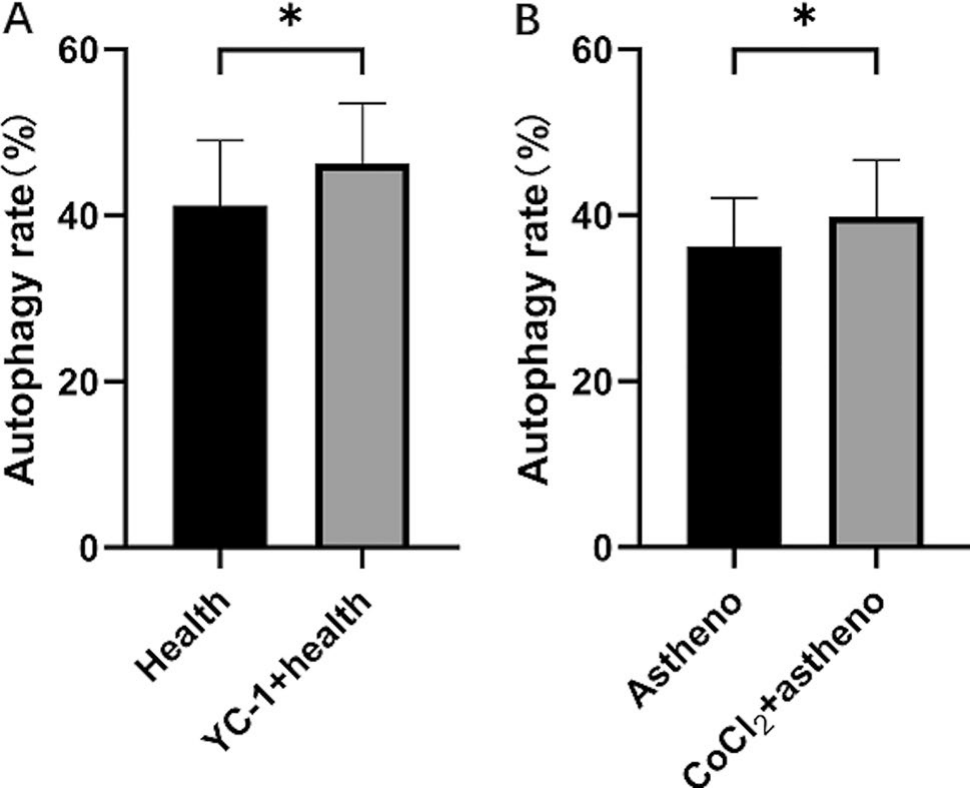
Effect of incubation in 5000 μM YC-1 (**A**, ^*^p < 0.05) or 100nM CoCl_2_ (**B**, ^*^p < 0.05) at 37 ℃ for 60 min on sperm autophagy level determined with MDC (Monodansylcadaverin) autophagy staining test kit.

Compared with the Health control group, the degree of sperm autophagy was significantly increased after 60 min of treatment with 5000 µM YC-1. Similarly, compared with the Astheno control group, the sperm autophagy rate was increased after 60 min of treatment with 100 nM CoCl_2_ (Fig. 2).

### Proteomics results

#### Protein and peptide content determination

A total of 12 label-free quantitative proteomics experiments were conducted on 3 samples from each of the 4 groups. The protein concentrations of all 4 groups were determined to be 1.50 ± 0.14 μg/μL, indicating a reasonable range of protein concentrations. SDS-PAGE gel electrophoresis analysis was performed on each group of protein samples. Every protein bands in the electrophoresis plates were clear and not obviously degraded, suggesting that the samples were suitable for further analysis.

### Differential protein screening

Distinct molecular peaks were observed in the total ion chromatography, and the main fragment ion peaks were found to be relevant to the protein quality.

In comparison to the Health group, the Astheno group showed 17 differentially expressed proteins, including 5 up-regulated and 12 down-regulated proteins. The YC-1 + health group exhibited 34 differentially expressed proteins compared to the Health group, with 1 up-regulated and 33 down-regulated proteins. Similarly, when compared with Astheno group, the CoCl_2_ + astheno group revealed 35 differentially expressed proteins, all which were down-regulated (Table 2).

**Table 2:** Summary of the differentially expressed proteins. Astheno is Asthenozoospermia. Values are number of protein. Semen of YC-1 + health group was incubated with 5000 μM YC-1 at 37 ℃ for 60 min. Semen of CoCl_2_ + astheno group was incubated with 100 nM CoCl_2_ at 37 ℃ for 60 min.

Group	Total number of differential protein	Up-regulated protein number	Down-regulated protein number
Astheno vs Health	17	5	12
(YC-1 + health) vs Health	34	1	33
(CoCl_2_ + astheno) vs Astheno	35	0	35

### Bioinformatics analysis on differentially expressed proteins

#### Clustering analysis

**Figure 3:**
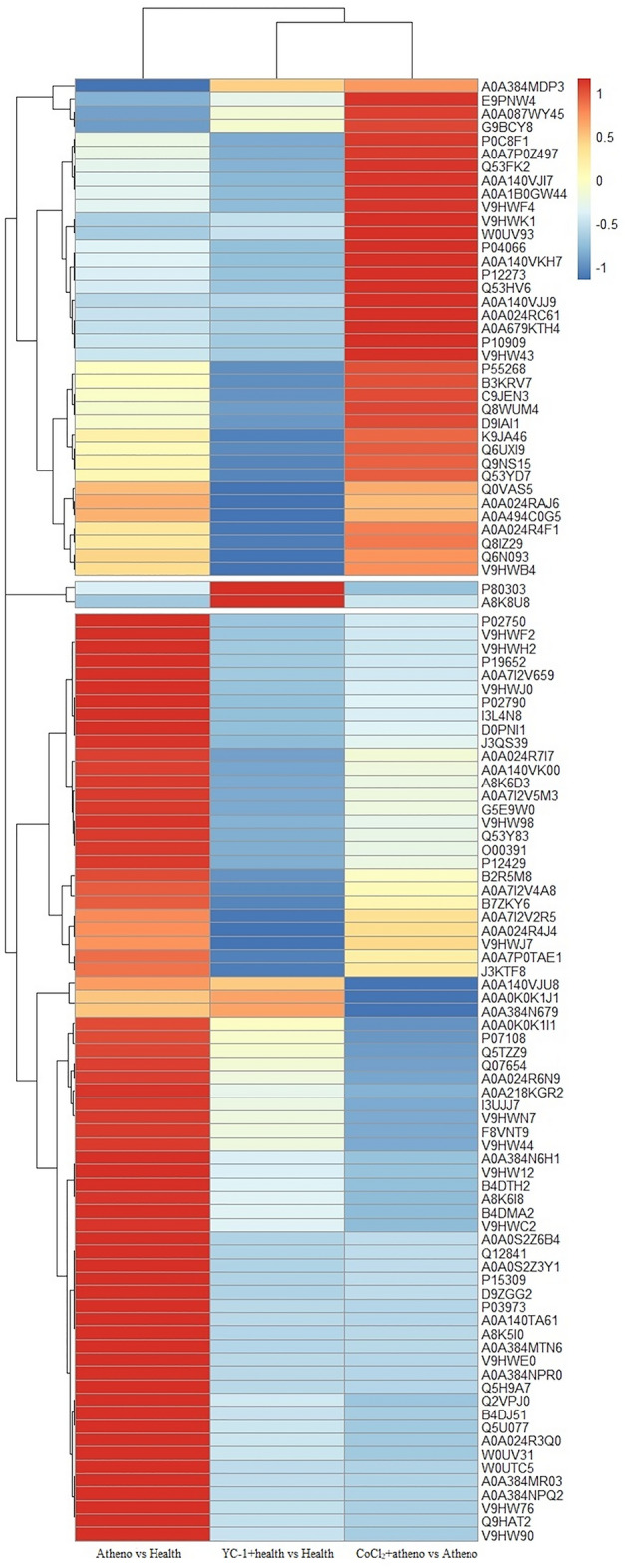
Heat-map of differentially abundant proteins of human semen groups.

The clustering heat map clearly indicated that the separation of differentially expressed proteins between the Astheno group and Health group, YC-1 + health group and Health group, as well as the CoCl_2_ + astheno group and Astheno group. This suggests the identified proteins represented significant inter-group differences of the semen samples (Fig. 3).

#### Gene ontology (GO) analysis

GO classification annotation (refer to Appendix 1 Fig. 1) revealed that the differentially expressed semen proteins in the Astheno group were primarily associated with IgA immunoglobulin complex, ubiquitin ligase complex, endoplasmic reticulum quality control compartment, and more. The molecular functions basically were binding and enzyme activity. The biological processes were mainly related to protein transport, protein localization, protein targeting and respiratory burst (refer to Appendix 1 Fig. 1A).

The differential proteins in the YC-1 + health group were mainly distributed in the endoplasmic reticulum exit site, presynaptic active zone, phosphopyruvate hydratase complex, bicellular tight junction and chloride channel complex. The molecular functions primarily included NADP binding and enzyme activity. The biological processes were involved in biosynthetic process and metabolic process (refer to Appendix 1 Fig. 1B).

The differential proteins in the CoCl_2_ + astheno group semen were primarily found in the endoplasmic reticulum lumen, extracellular matrix, lysosome lumen, vacuolar membrane and cytoplasmic vesicle lumen. The molecular function included protein binding, enzyme activity and extracellular matrix structural constituent. The biological process was associated with metabolic process, negative regulation of enzyme activity, glycolytic process and positive regulation of oxidative stress-induced cell death (refer to Appendix 1 Fig. 1C).

KEGG pathway enrichment analysis (www.kegg.jp/kegg/kegg1.html) (refer to Appendix 2 Table 1).

The differentially expressed human semen proteins in the Astheno group were found to be involved in various metabolic pathways, including protein processing in endoplasmic reticulum, Th17 cell differentiation, progesterone-mediated oocyte maturation, and more.

The differential proteins in the YC-1 + health group primarily participated in biosynthesis of amino acids, metabolism, glycolysis/gluconeogenesis, HIF-1 signaling pathway, and other pathways.

The differential proteins in the CoCl_2_ + astheno group mainly contributed to glycolysis/gluconeogenesis, biosynthesis of amino acids and fatty acid, carbon metabolism, complement and coagulation cascades, HIF-1 signaling pathway, PI3K-Akt signaling pathway, and more.

#### Protein–protein interaction (PPI) network analysis (https://www.uniprot.org)

**Figure 4:**
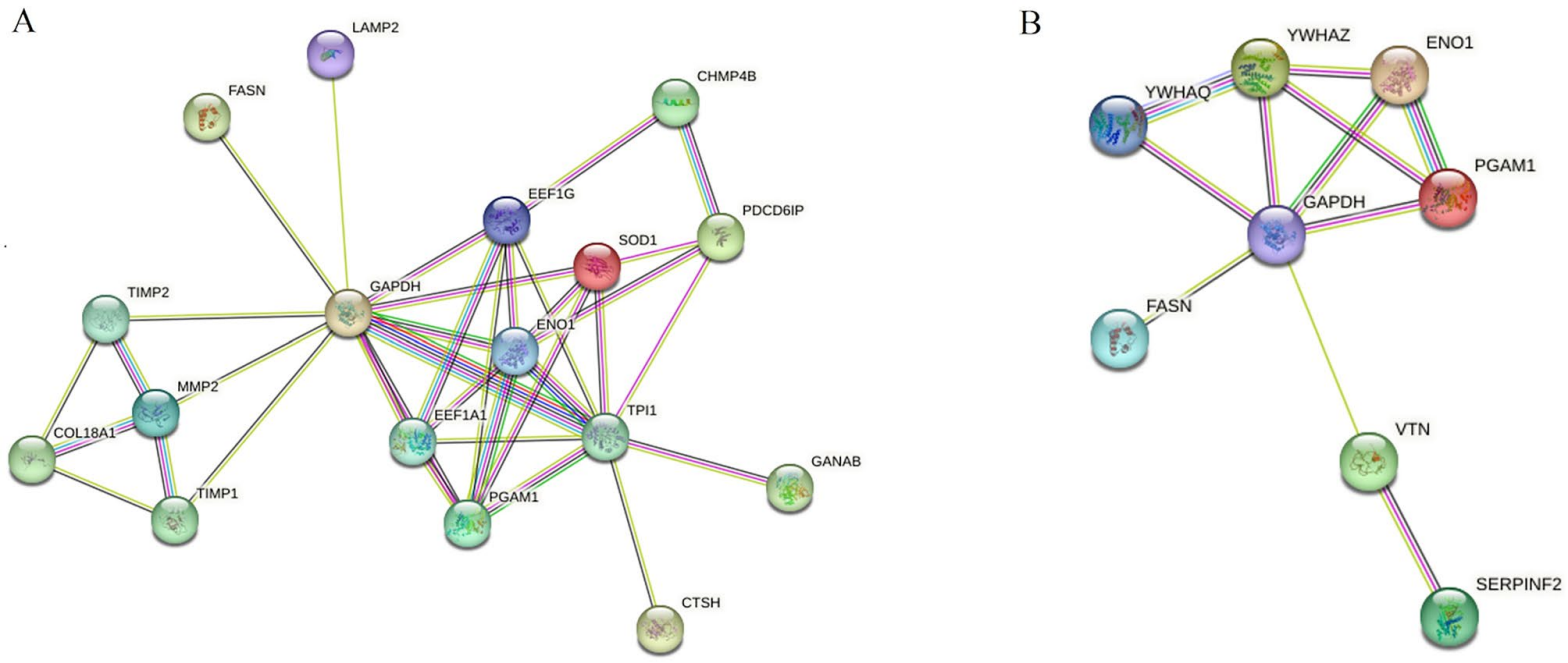
Protein–protein interactions (PPIs) of differentially abundant proteins in human semen after STRING analysis on YC-1 + health group (**A**) and in CoCl_2_ + astheno group (**B**).

According to STRING analysis, the PPI of differential proteins in the YC-1 + health group (Fig. 4A) was primarily associated with glycolysis/gluconeogenesis, biosynthesis of amino acids, carbon metabolism, HIF-1 signaling pathway and others. The proteins were mainly expressed in semen and lymph.

The PPI in the CoCl_2_ + astheno group (Fig. 4B) was mainly involved in the biological processes such as positive regulation of protein insertion into the mitochondrial membrane, apoptotic signaling pathways, canonical glycolysis, regulation of plasminogen activation, gluconeogenesis, negative regulation of coagulation and more. The proteins were associated with pathways including glycolysis/gluconeogenesis, biosynthesis of amino acids, HIF-1 signaling pathway and PI3K/AKT signaling pathway.

The PPIs observed were consistent with GO and KEGG pathway annotations.

#### Joint analysis of differential proteins between groups

The intersection proteins of differentially expressed proteins in the YC-1 + health group, CoCl_2_ + astheno group, and Astheno group were integrated to identify the up-regulated or down-regulated trends after reagent interference. Compared with the Health group, the Q0VAS5 protein (Histone H4, His H4) was up-regulated in the YC-1 + health group, while A0A7I2V5M3 protein (Cathepsin L, Cath L), E9PKT6 protein (Cathepsin H), P58499 protein (Protein FAM3B), Q53YD7 protein (Elongation factor 1-gamma) were down-regulated. These differences resulted in an increased level of sperm autophagy and decreased sperm motility. Furthermore, in comparison with the Health group, the A0A2R8Y430 protein (Glutathione synthetase, Glu), Q9UM22 protein (Mammalian ependymin-related protein 1), Q9Y6R7 protein (IgGFc-binding protein), P58499 protein (Protein FAM3B), Q13438 protein (Protein OS-9) were down-regulated in the CoCl_2_ + astheno group. This suggests that CoCl_2_ inhibited the expression of these proteins, leading to an and enhanced level of sperm autophagy and reduction in sperm motility.

### Expression levels of selected proteins in human semen

The expression levels of related proteins are presented in Table 3. HIF-1αand LC3B exhibited high expression in both the YC-1 + health group and the CoCl_2_ + astheno group. Compared with the Health group, the level of glycolysis-related protein enolase 1 (ENO1) did not change significantly in the YC-1 + health group. However, compared to the Astheno group, the ENO1 level was higher in the CoCl_2_ + astheno group. The levels of the glycolysis-related protein PGK1 did not differ between the Health group and the YC-1 + health group, as well as between the Astheno group and the CoCl_2_ + astheno group. Additionally, in comparison to the Health group, the A0A2R8Y430 protein (Glutathione synthetase) was down-regulated in the CoCl_2_ + astheno group, the A0A7I2V5M3 protein (Cathepsin L) was down-regulated, and the Q0VAS5 protein (Histone H4) was highly expressed in the YC-1 + health group. The levels of HIF-1α, LC3B, ENO1, and PGK1 in the Astheno group differed significantly from those in the Health group.

**Table 3:** Effects of HIF-1α interfernts on levels of related proteins in human semen.

	HIF-1α (ng/mL)	LC3B (nmol/L)	ENO1 (nmol/L)	PGK1 (ng/mL)	Cathepsin L (pg/mL)	Glutathione synthetase (nmol/L)	Histone H4 (ng/10^6^ sperm)
Health group	1.9 ± 0.7	3.5 ± 1.9	7.2 ± 2.2	49.4 ± 14.7	2998 ± 893	7.3 ± 5.4	139 ± 46
YC-1 + health group	3.7 ± 1.3^+^	4.7 ± 1.3^++^	6.8 ± 2.6	52.3 ± 21.6	1932 ± 534^++^	6.7 ± 6.1	156 ± 45^++^
Astheno group	2.3 ± 0.9	6.4 ± 2.1^++^	1.9 ± 0.5^++^	34 ± 7.2^++^	3143 ± 967	6.5 ± 5.6	147 ± 70
CoCl_2_ + astheno group	3.5 ± 0.8**	8.9 ± 3.3**	3.4 ± 1.3**	37 ± 7.2	3226 ± 1273	6.9 ± 5.7	236 ± 57**

Values are mean ± SD. Analysis of variance (ANOVA) has been used. Compared with the Health group, ^+^p < 0.05, ^++^p < 0.01; Compared with the Astheno group, **p < 0.01.

## Discussion

### Relationship between semen HIF-1α and sperm autophagy in human

In this study, we investigated the relationship between semen HIF-1α and sperm autophagy in humans. We found that incubating human semen with HIF-1α disruptors, such as 5000μΜ YC-1 or 100nM CoCl_2,_ resulted in an increase in HIF-1α level and sperm autophagy rate, as well as a decrease in progressive sperm motility. Previous studies have reported that patients with asthenozoospermia or varicocele exhibited significantly lower sperm motility and elevated HIF-1α levels^22,23^. Furthermore, both mouse experiment data and human clinical data have shown that sperm motility and density are significantly reduced under hypoxic conditions compared to normoxic conditions^24–26^. It has been observed that hypoxia-induced overexpression of HIF-1α triggers the activation of autophagy pathway^27,28^, while knockout of HIF-1α inhibits the expression of mitochondrial autophagy-related genes^29^. In this study, when human semen was incubated with 5000 µM YC-1 or 100 nM CoCl_2_ for 60 min, we observed increased levels of LC3B in both YC-1 + health group and CoCl_2_ + astheno group, indicating a significant increase in sperm autophagy.

The energy required for sperm motility primarily comes from glycolysis and gluconeogenesis. In certain cell types, such as the granulosa cells of mice, HIF-1α-dependent autophagy has been shown to play a protective role in facilitating the switch to glycolysis, which is important for energy supply and cell survival^30^. Improving the function of sperm mitochondrial oxidative respiratory chain could increase ATP levels and significantly enhance the rate of forward-moving sperm^31–33^. Inhibition of glycolysis in rats has been found to dramatically reduce sperm motility and fertility^34^, and this reduction is closely correlated with HIF-1α levels^35^. In the pathway analysis conducted in this study, the differentially expressed proteins in the YC-1 + health group or CoCl_2_ + astheno group were found to be enriched in various pathways, including the HIF-1 signaling pathway, glycolysis/gluconeogenesis signaling pathway, amino acid biosynthesis, and other metabolic processes. The reduction in sperm motility observed in both groups suggests that hypoxia regulates sperm autophagy and motility through these signaling pathways. This finding is consistent with previous research that highlighted the pronounced involvement of the hypoxia pathway in asthenozoospermia^16^.

YC-1 is an inhibitor of HIF-1α and is known to block the expression of HIF-1α under hypoxia conditions, thereby inhibiting its transcriptional activity. However, in this study, we observed that YC-1 did not reduce the level of HIF-1α. On the contrary, HIF-1α levels increased in the YC-1 + health group. This unexpected result may be attributed to YC-1 increasing the stability of HIF-1α rather than inhibiting its expression^36^. Consequently, HIF-1α accumulated to high level, leading to an increase of the autophagy rate.

### Differentially expressed autophagy-related proteins in asthenozoospermia

The expression of various proteins is closely linked to the metabolic processes and physiological mechanisms within organisms. In this particular study, we observed distinct expression patterns in the semen samples of the YC-1 + health group and the CoCl_2_ + astheno group. Specifically, we found that the protein Q0VAS5 (Histone H4) was upregulated, while the protein A0A7I2V5M3 (Cathepsin L), E9PKT6, P58499, and Q53YD7 were downregulated in the semen samples of YC-1 + health group. Similarly, the protein A0A2R8Y430 (Glutathione synthetase), Q9UM22, Q9Y6R7, P58499, and Q13438 were downregulated in the CoCl_2_ + astheno group. These observed changes in protein expression align with the alterations typically observed in cases of asthenozoospermia.

Histone H4 is a critical components of nucleosomes, and modifications such as acetylation or methylation of histone H4 have been shown to impact sperm morphology and motility^37^. Studies have demonstrated that impaired exchange of histones with protamine can reduce fertilization^38^.

Cathepsin L is an enzyme that degrades proteins in autophagosomes, inhibit the expression of lysosomal enzyme, and promote autophagy. Interestingly, in the YC-1 + health group, activation of Cathepsin L protein decreased, but autophagy was unexpectedly stimulated. This could be attributed to a negative feedback loop, where autophagy suppresses Cathepsin L activity. Additionally, Cathepsin L is known to be a target of HIF-1α. In the YC-1 + health group, the increased levels of HIF-1α may be a mechanism to maintain low autophagy rate and cellular homeostasis^39^.

Glutathione synthetase is responsible for synthesizing the antioxidant glutathione (GSH) in cells and plays a role in intracellular oxidation and antioxidant processes. Autophagy is induced in response to oxidative stress to alleviate damage and protect cell survival. Reactive oxygen species (ROS) are important for sperm capacitation, but an imbalance between ROS production and antioxidants can lead to oxidative stress and impair sperm function^40^. Studies have showed that high levels of glutathione in rat semen are associated with increased sperm motility, sperm concentration and sperm viability^41^.

Enolase 1 (ENO1) is involved in glycolysis and has a negative correlation with sperm motility^42^. These proteins are related to sperm autophagy and vitality, and may serve as potential biological markers or treatment targets for conditions characterized by low sperm motility such as asthenozoospermia.

In summary, our findings shed light on the role of specific proteins and signaling pathways in sperm autophagy and propose their potential as biomarkers for diagnosing and studying asthenozoospermia. However, it is important to note that the strength of the discoveries as evidence is limited by the use of YC-1 only in healthy patients and CoCl_2_ only in astheno patients in the present study. In order to obtain a more comprehensive understanding of the subject and provide stronger evidence in the field, our future research would include a wider range of doses and incorporate diverse subject groups by exploring additional interventions.

## Conclusions

Hypoxia was found to increase the level of autophagy in sperm through the activation of the HIF-1 signaling pathway and the glycolysis/gluconeogenesis signaling pathway.

Histone H4, cathepsin L, glutathione synthetase, and ENO1 emerged as potential biomarkers for asthenozoospermia.

### Supplementary Information

Supplementary Legends.

Supplementary Information 1.

Supplementary Information 2.

## Data Availability

The raw data of the study can be obtained from the corresponding author on enquiry.
