# Supplementary material for: Hypoxia enhances autophagy level of human sperms
Source: Sci Rep. 2024 Apr 11;14:8465. doi: 10.1038/s41598-024-59213-1 (PMC11009268; doi:10.1038/s41598-024-59213-1)
Supplement: Supplementary file 1 — Supplementary Legends. [file 41598_2024_59213_MOESM1_ESM.docx]

**Supplementary material**

Supplementary data to this article can be found online.

Additional file 1: Appendix 1 Figure 1, Histogram of GO analysis on differential proteins of human semen in Astheno group (**A**), YC-1+health (**B**) and CoCl_2_+astheno group (**C**).

Additional file 2: Appendix 2 Table 1**,** Main metabolic pathways involved by differential proteins of human semen.
