# Supplementary figures and images for: Hypoxia enhances autophagy level of human sperms
Source: Sci Rep. 2024 Apr 11;14:8465. doi: 10.1038/s41598-024-59213-1 (PMC11009268; doi:10.1038/s41598-024-59213-1)

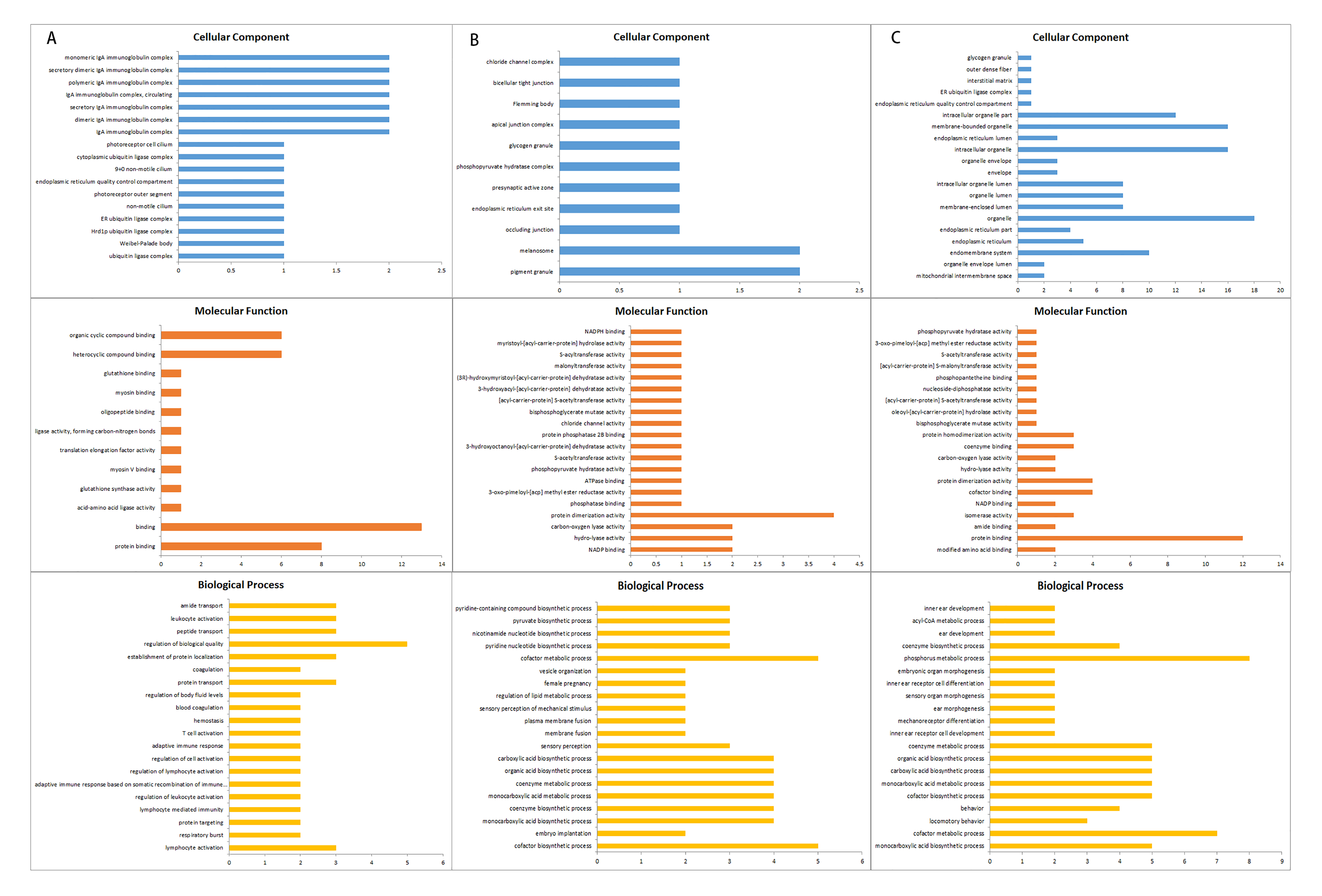

Supplement: Supplementary file 2 — Supplementary Information 1. [file 41598_2024_59213_MOESM2_ESM.tif]
