# Supplementary material for: Hypoxia enhances autophagy level of human sperms
Source: Sci Rep. 2024 Apr 11;14:8465. doi: 10.1038/s41598-024-59213-1 (PMC11009268; doi:10.1038/s41598-024-59213-1)
Supplement: Supplementary file 3 — Supplementary Information 2. [file 41598_2024_59213_MOESM3_ESM.docx]

| Pathway_acc | Pathway_name | P-value | Protein list |  |
| --- | --- | --- | --- | --- |
| **Astheno *vs* Health** | | | | |
| hsa04141 | Protein processing in endoplasmic reticulum | 0.0112635 | K9JA46,Q13438 |  |
| hsa04659 | Th17 cell differentiation | 0.01895206 | K9JA46 |  |
| hsa04914 | Progesterone-mediated oocyte maturation | 0.0375657 | K9JA46 |  |
| **YC-1+health *vs* Health** | | | | |
| hsa01230 | Biosynthesis of amino acids | 0.003580911 | A0A024R4F1,Q6FHU2,V9HVZ4 |  |
| hsa01200 | Carbon metabolism | 0.008670311 | A0A024R4F1,Q6FHU2,V9HVZ4 |  |
| hsa01100 | Metabolic pathways | 0.01827896 | A0A024R4F1,Q6FHU2,V9HWJ0,P49327,V9HVZ4,Q53H01 |  |
| hsa05230 | Central carbon metabolism in cancer | 0.01902029 | Q6FHU2,Q6FHU2 |  |
| hsa00010 | Glycolysis / Gluconeogenesis | 0.01988376 | A0A024R4F1,Q6FHU2,V9HVZ4 |  |
| hsa04066 | HIF-1 signaling pathway | 0.02599943 | A0A024R4F1,V9HVZ4 |  |
| hsa04146 | Peroxisome | 0.03790412 | V9HWC9 |  |
| hsa04911 | Insulin secretion | 0.03790412 | A0A024R7I7 |  |
| hsa01212 | Fatty acid metabolism | 0.03790412 | P49327 |  |
| hsa00061 | Fatty acid biosynthesis | 0.03790412 | P49327 |  |
| hsa00260 | Glycine, serine and threonine metabolism | 0.03790412 | Q6FHU2 |  |
| hsa01522 | Endocrine resistance | 0.03790412 | A0A024R6R4 |  |
| **CoCl_2_+astheno *vs* Astheno** | | | | |
| hsa00010 | Glycolysis / Gluconeogenesis | 0.000451567 | V9HVZ4,A0A024R4F1, Q6FHU2 |  |
| hsa01230 | Biosynthesis of amino acids | 0.000629142 | V9HVZ4,A0A024R4F1, Q6FHU2 |  |
| hsa01200 | Carbon metabolism | 0.002170374 | V9HVZ4,A0A024R4F1, Q6FHU2 |  |
| hsa04610 | Complement and coagulation cascades | 0.016359648 | P08697, D9ZGG2 |  |
| hsa04066 | HIF-1 signaling pathway | 0.025532271 | V9HVZ4,A0A024R4F1 |  |
| hsa04110 | Cell cycle | 0.033866998 | P27348, D0PNI1 |  |
| hsa04114 | Oocyte meiosis | 0.035847248 | P27348, D0PNI1 |  |
| hsa04142 | Lysosome | 0.036349315 | Q53H01,A0A140VJE4 |  |
| hsa00061 | Fatty acid biosynthesis | 0.040679531 | P49327 |  |
| hsa00531 | Glycosaminoglycan degradation | 0.042892899 | A0A140VJE4 |  |
| hsa04151 | PI3K-Akt signaling pathway | 0.045537444 | P27348,D9ZGG2,D0PNI1 |  |
| hsa04390 | Hippo signaling pathway | 0.049766774 | P27348, D0PNI1 |  |
| hsa05160 | Hepatitis C | 0.049766774 | P27348, D0PNI1 |  |

**Appendix 2 Table 1.** Main metabolic pathways involved to differential proteins of human semen. Astheno is Asthenozoospermia. Semen of YC-1+health group and semen of CoCl_2_+astheno group were incubated with 5000μM YC-1 or 100nM CoCl_2_, respectively, at 37℃ for 60 minutes.
